# Characterization of Low-Loss Dielectric Materials for High-Speed and High-Frequency Applications

**DOI:** 10.3390/ma15072396

**Published:** 2022-03-24

**Authors:** Tzu-Nien Lee, John-H Lau, Cheng-Ta Ko, Tim Xia, Eagle Lin, Kai-Ming Yang, Puru-Bruce Lin, Chia-Yu Peng, Leo Chang, Jia-Shiang Chen, Yi-Hsiu Fang, Li-Yueh Liao, Edward Charn, Jason Wang, Tzyy-Jang Tseng

**Affiliations:** Unimicron Technology Corporation, No. 179, Shanying Road, Taoyuan City 33341, Taiwan; zn_lee@unimicron.com (T.-N.L.); ct_ko@unimicron.com (C.-T.K.); tim_xia@unimicron.com (T.X.); eagle.lin@unimicron.com (E.L.); henryyang@unimicron.com (K.-M.Y.); bruce_lin@unimicron.com (P.-B.L.); tony_peng@unimicron.com (C.-Y.P.); leo_chang@unimicron.com (L.C.); js_chen@unimicron.com (J.-S.C.); yihsiu_fang@unimicron.com (Y.-H.F.); lisa_liao@unimicron.com (L.-Y.L.); edwardcharn@unimicron.com (E.C.); jason_wang1@unimicron.com (J.W.); tjtseng@unimicron.com (T.-J.T.)

**Keywords:** dielectric materials, high-speed and high-frequency, insertion loss and return loss

## Abstract

In this study, the Df (dissipation factor or loss tangent) and Dk (dielectric constant or permittivity) of the low-loss dielectric material from three different vendors are measured by the Fabry–Perot open resonator (FPOR) technique. Emphasis is placed on the sample preparation, data collection, and the comparison with the data sheet values provided from vendors. A coplanar waveguide with ground (CPWG) test vehicle with one of these raw dielectric materials (vendor 1) is designed (through Polar and simulation) and fabricated. The impedance of the test vehicle is measured by TDR (time-domain reflectometer), and the effective Dk of the test vehicle is calculated by the real cross-section of the metal line width, spacing, and thickness of the test vehicle and a closed-form equation. In parallel, the insertion loss and return loss are measured with the VNA (vector network analyzer) of the test vehicle. Finally, the measurement and simulation results are correlated. Some recommendations on the low-loss dielectric materials of the Dk and Df are also provided.

## 1. Introduction

The semiconductor industry has identified five major growth engines (applications): (1) mobile, such as smartphones, smartwatches, notebooks, wearables, tablets, etc., [1,2,3,4]; (2) high-performance computing (HPC), also known as supercomputing, which is able to process data and perform complex calculations at high speeds on a supercomputer [5,6]; (3) autonomous vehicle (or self-driving cars) [7]; (4) IoT (internet of things), such as smart factories and smart health; [3,8,9] and (5) big data (for cloud computing) and instant data (for edge computing) [5,6]. The system-technology drivers such as 5G (fifth generation technology standard for broadband cellular networks) are boosting the growth of these five semiconductor applications.

According to the US Federal Communications Commission: (a) the mid-band spectrum (also called Sub-6 GHz 5G) is defined as 900 MHz < frequency < 6 GHz and data speeds ≦ 1 Gbps; and (b) the high-band spectrum (also called 5G millimeter wave or 5G mmWave) is defined as 24 GHz ≦ frequency ≦ 100 GHz and 1 Gbps < data speeds ≦ 10 Gbps. In order to meet the requirements for boosting signal transmission speed/rate and managing a huge data flood, advanced development of semiconductors, packaging, materials, etc. is necessary. With respect to the electrical performance of insulation materials, low-loss Df (dissipation factor or loss tangent) and Dk (dielectric constant or permittivity) materials are highly preferred for 5G applications [10,11]. The recent results of the preparation and investigation of substituted ferrites, promising for microwave applications, have been published in [12,13]. Additionally, the combination of different compounds with excellent electronic properties leads to new composite materials which have earned great technological interest in recent years. The addition of a second phase can significantly improve the electronic properties of the resulting composite material, as shown in [14,15].

The following equation shows the transmission loss, which is equal to the sum of the conductor loss and dielectric loss. Conductor loss is proportional to the conductor skin resistance and the square root of Dk. Usually, the higher the frequency, the closer to the conductor surface the current signal flows (skin effect). For a rough surface conductor, the current signal is presumed to travel a longer distance on the surface, which leads to greater transmission loss. Thus, utilizing copper with lower surface roughness can reduce the conductor skin resistance. (Conductor loss is outside the scope of this paper.) The dielectric loss is proportional to the frequency, Df, and the square root of Dk. Thus, in order to achieve lower transmission loss, lower values of Df and Dk are needed [2,10,16,17].

Transmission Loss=Conductor Loss+Dielectric Loss

$$\mathrm{Conductor}\;\mathrm{Loss}\approx \mathrm{Conductor}\;\mathrm{Skin}\;\mathrm{Resistance}\;\times \sqrt{D_{k}}$$

$$\mathrm{Dielectric}\;\mathrm{Loss}\approx f\;\times {\;D}_{f}\times \sqrt{D_{k}}$$

where

f=Frequency

$$D_{f}=\mathrm{Dissipation}\;\mathrm{Factor}\;\left(\mathrm{Loss}\;\mathrm{Tan}\mathrm{gent}\right)$$

$$D_{k}=\mathrm{Dielectric}\;\mathrm{Constant}\;\left(\mathrm{Perimittivity}\right)$$

In this study, the Dk and Df of three different raw dielectric materials are characterized by the Fabry–Perot open resonator (FPOR) measurement technique [18]. The sample preparation is based on IEC 61189:2015. These values are compared with those from the data sheets of the raw materials, and the difference will be discussed.

**Figure 1:**
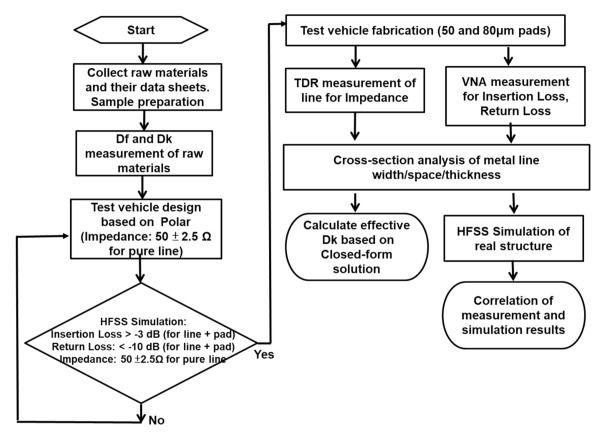
Flowchart of dielectric materials characterization.

With the help of Polar and ANSYS’ HFSS (high-frequency structure simulator) software, a coplanar waveguide with ground (CPWG) test vehicle with one of these raw dielectric materials (vendor 1) is designed and fabricated, as shown in Figure 1. Then, the impedance of the test vehicle is measured by TDR (time-domain reflectometer), and the effective Dk of the test vehicle is calculated through a closed-form equation and the real cross-section of the metal line width, spacing, and thickness as shown in Figure 1. Separately, the insertion loss and return loss are measured with the VNA (vector network analyzer) of the test vehicle with pads. Finally, the measurement and simulation results are correlated.

## 2. Raw Materials and Their Data Sheets

The raw materials data sheets of three different vendors are shown in Table 1, where their Dk, Df, and other important physical and mechanical material properties are also provided. It can be seen that: (a) for vendor 1, it is a BCB (benzocyclobutene) polymer with a curing temperature of 170 °C or 200 °C, and its Dk and Df are, respectively, 2.66 and 0.0031 at 28.3 GHz and 2.64 and 0.0032 at 39.6 GHz; (b) for Vendor 2, it is a PPE (polyphenylene ether) polymer with a curing temperature of 200 °C, and its Dk and Df are, respectively, 2.48 and 0.003 at 28 GHz and 2.57 and 0.003 at 40 GHz; and (c) for vendor 3, it is a PI (polyimide) polymer with a curing temperature of 230 °C, and its Dk and Df are, respectively, 3.07 and 0.01 at 19.36 GHz, 3.11 and 0.01 at 29.1 GHz, and 2.9 and 0.01 at 38.9 GHz.

## 3. Sample Preparation

**Figure 2:**
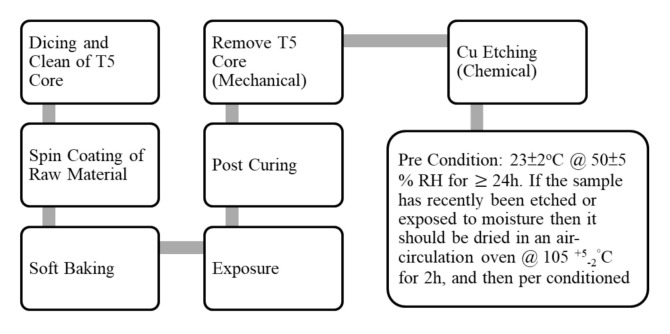
Sample preparation procedure for the measurement.

**Figure 3:**
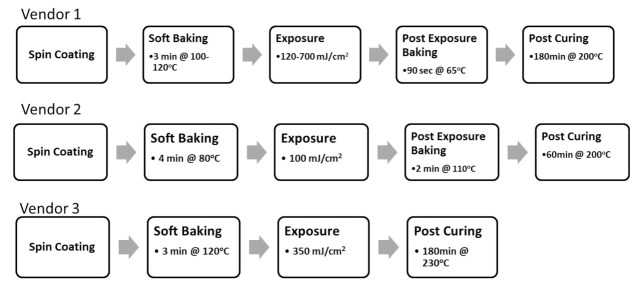
Sample preparation condition for vendors.

**Figure 4:**
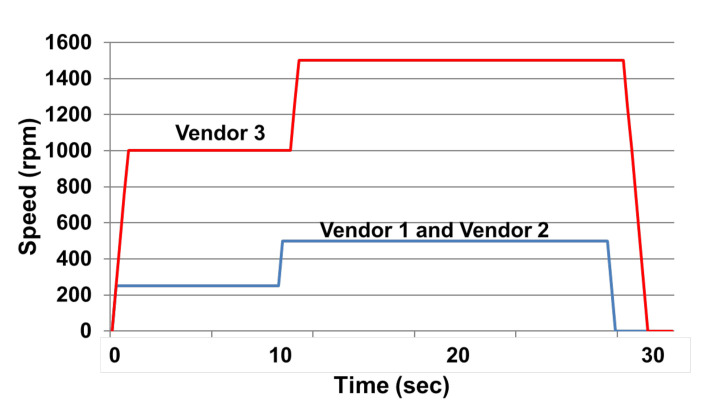
Speed vs. time.

The sample preparation procedure is based on the guidance of IEC 61189:2015, which is basically shown in Figure 2, and the sample preparation conditions are recommended by the vendors, as shown in Figure 3. It can be seen that, in this study, we use a T5 core panel to let the raw materials, PID (photoimageable dielectric), to be spun on. The spin coating condition for each vendor is shown in Figure 4. It can be seen that for vendors 1 and 2, the initial speed is 250 rpm for 10 s and then 500 rpm for 20 s, and for vendor 3, the initial speed is 1000 rpm for 10 s and 1500 rpm for 20 s.

**Figure 5:**
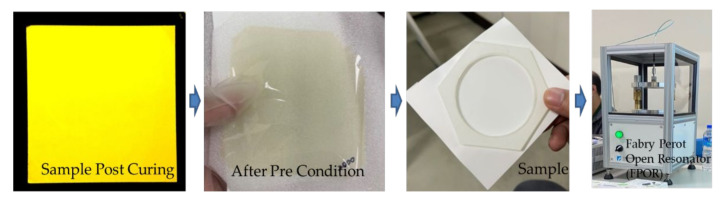
Fabry–Perot open resonator (FPOR).

The resulting sample thickness (Table 2) for vendor 1 is 28 μm, for vendor 2 is 57 μm, and for vendor 3 is 17 μm. There are at least two reasons for the difference in sample thickness: (a) different viscosity—the higher, the thicker; and (b) different spin coating speed—the faster, the thinner. After the sample preparation procedure (Figure 2) and condition (Figure 3) for vendors 1, 2, and 3, the typical images of after post-curing and after pre-conditioning are shown in Figure 5.

## 4. Fabry-Perot Open Resonator (FPOR)

The Fabry-Perot open resonator (FPOR) measurement technique is adopted in this study (Figure 5). It can measure the sample sizes of 10 cm × 10 cm × 10 μm to 2 mm with a spot size of 5 cm in diameter and the Dk and Df from 20 to 44 GHz. The ambient test temperature should be (23 ± 2) °C. The variation should not exceed 1 °C during the test. Furthermore, the data are taken from at least 11 points on the sample.

### 4.1. FPOR Measurement Results of Vendor 1

**Figure 6:**
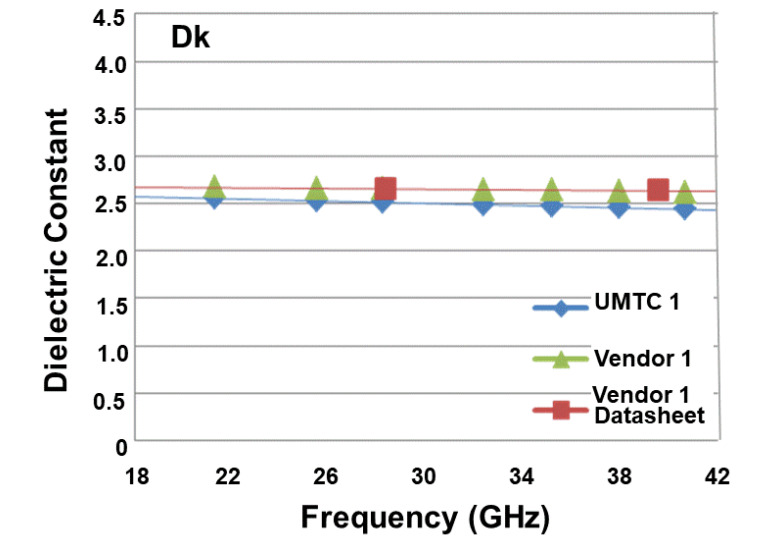

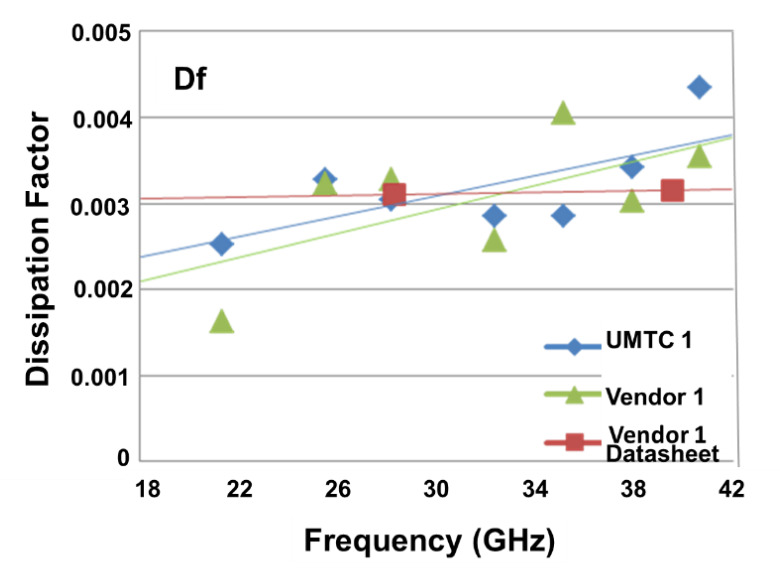
Dk and Df of Vendor 1.

Table 3 tabulates the data sheet values and measurement results (Dk and Df) of vendor 1′s low-loss dielectric material for various frequencies. In this table, it shows: (a) the Dk and Df measured from the sample that we made (UMTC 1) and the sample provided by the vendor (vendor 1) and the Dk and Df from the data sheet of cendor 1 (and all of these are summarized in Figure 6); and (b) the percent deviation in Dk and Df. It can be seen that: (a) for Dk, the results from UMTC 1 (2.51 at 28.2 GHz and 2.46 at 38 GHz) are very close to those from vendor 1 (2.653 at 28.2 GHz and 2.62 at 38); (b) additionally, for Dk, the results from UMTC 1 are very close to those from the data sheet of vendor 1 (2.66 at 28.3 GHz and 2.64 at 39.6 GHz); (c) for Df, the results from UMTC 1 (0.003 at 28.2 GHz and 0.0034 at 38 GHz) are very close to those from vendor 1 (0.00328 at 28.2 GHz and 0.00302 at 38 GHz) are very close to those from vendor 1 (2.59 at 28.2 GHz and 2.62 at 38 GHz); and (d) also for Df, the results from UMTC 1 are very close to those from the data sheet of vendor 1 (0.0031 at 28.3 GHz and 0.0032 at 39.6 GHz). The trend of Dk is independent of the frequency; however, the trend of Df is frequency-dependent—the higher the frequency, the higher the Df.

### 4.2. FPOR Measurement Results of Vendor 2

**Figure 7:**
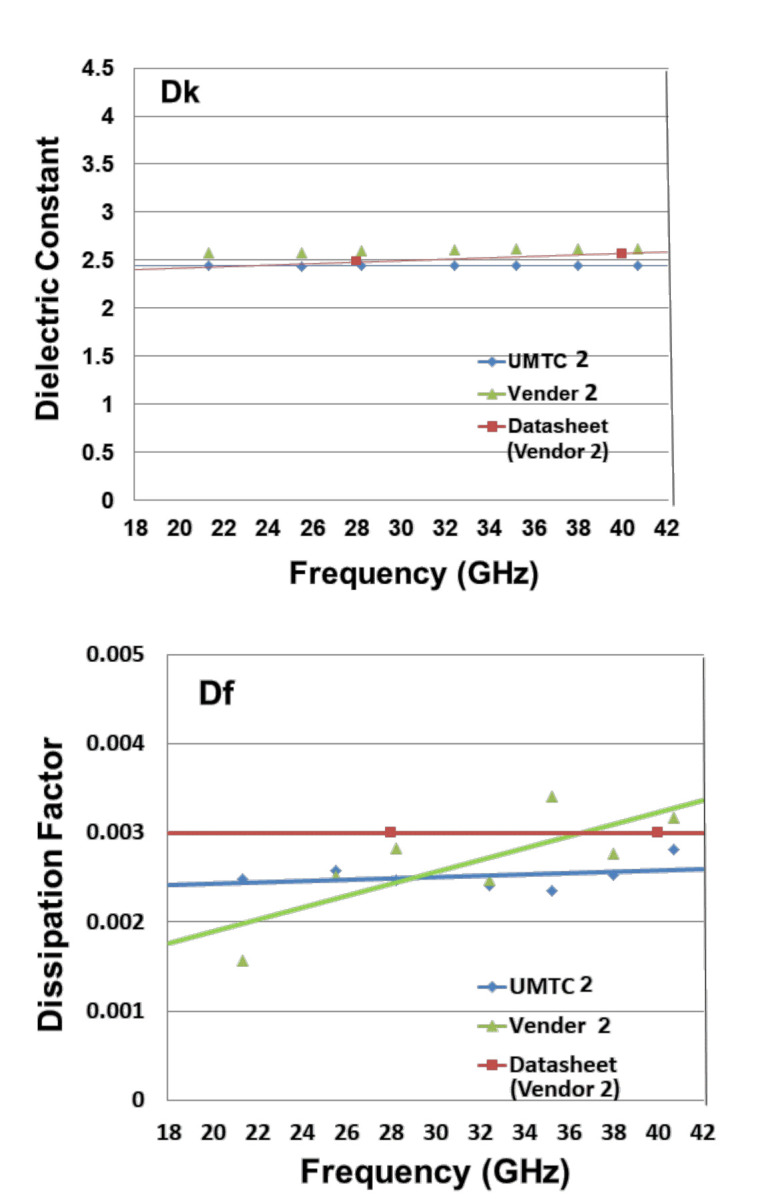
Dk and Df of Vendor 2.

Table 4 tabulates the data sheet values and measurement results (Dk and Df) of the low-loss dielectric material from vendor 2 for various frequencies (Figure 7). It can be seen that: (a) for Dk, the results from UMTC 2 (2.4719 at 28.2 GHz and 2.4705 at 38 GHz) are very close to those from vendor 2 (2.59 at 28.2 GHz and 2.62 at 38); (b) additionally, for Dk, the results from UMTC 2 are very close to those from the data sheet of vendor 2 (2.48 at 28 GHz); (c) for Df, the results from UMTC 2 (0.00247 at 28.2 GHz and 0.00262 at 38 GHz) are reasonably close to those from vendor 2 (0.00282 at 28.2 GHz and 0.00277 at 38 GHz); and (d) also for Df, the results from UMTC 2 are reasonably close to those from the data sheet of vendor 2 (0.003 at 28 GHz). Again, the trend in Dk is basically independent of the frequency. On the other hand, the trend in Df is to be higher for higher frequencies.

### 4.3. FPOR Measurement Results of Vendor 3

**Figure 8:**
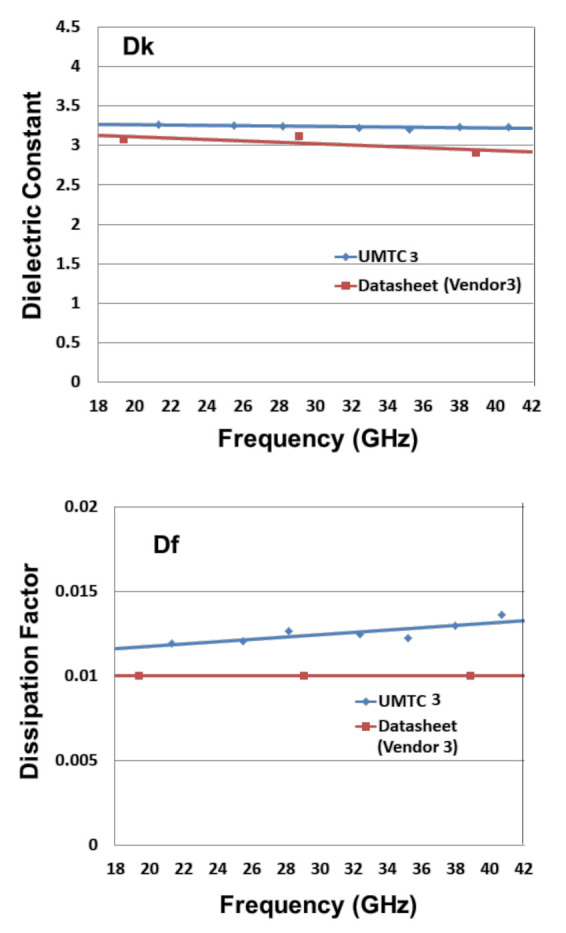
Dk and Df of Vendor 3.

Table 5 tabulates the data sheet values and measurement results (Dk and Df) of the low-loss dielectric material from vendor 3 for various frequencies (Figure 8). It can be seen that: (a) for Dk, the measurement results from UMTC 3 (3.26 at 21.3 GHz, 3.24 at 28.2 GHz, and 3.23 at 40.7 GHz) are very close to those from the data sheet of vendor 3 (3.07 at 19.36 GHz, 3.11 at 29.1 GHz, and 2.9 at 38.9 GHz); and (b) for Df, the results from UMTC 3 (0.0119 at 21.3 GHz, 0.0127 at 28.2 GHz, and 0.0136 at 40.7 GHz) are reasonably close to those from data sheet of vendor 3 (0.01 at 19.36 GHz, 0.01 at 29.1 GHz, and 0.01 at 38.9 GHz). Again, Dk is frequency-independent, and Df is frequency-dependent—the higher the frequency, the higher the Df.

### 4.4. Comparison between the Measurement Results from Vendors

With the frequencies under consideration (up to 40 GHz) in this study, the ranges of measurement results of Dk from the dielectric materials provided by vendor 1 and vendor 2 are within 2.45 and 2.67 and of Df are within 0.0025–0.004. These values of Dk and Df agree (in the same ballpark) with most of the values published. These materials are made from BCB and PPE, with a curing temperature ≦ 200 °C. On the other hand, the measurement results of Dk (3.2–3.26) and Df (0.0119–0.0136) from the dielectric material provided by vendor 3 are on the high side, especially the Df, which is a few times higher than those of vendors 1 and 2. The material of vendor 3 is made from PI, with a curing temperature of 230 °C.

According to the above measurement results, the BCB and PPE samples show better performance in the electrical material properties (Dk and Df) and repeatability. In contrast, the PI sample shows the worst repeatability and electrical material properties. The Dk and Df measurement results may be affected by environment, measuring instrument, and sample fabrication flow. According to Table 1, the PID, which is PI-based, shows the highest moisture absorption (about 2.23%). In other words, the PI-based samples are easily affected by the environment. The BCB- or PPE-based materials are suitable for the following test vehicle fabrication.

## 5. Test Vehicle Designed by Polar and ANSYS

### 5.1. Test Vehicle Designed by Polar

The dimensions of coplanar waveguide with ground (CPWG) are designed by Polar design: the dielectric height = 7 μm; dielectric constant of vendor 1 = 2.66; trace width = 15 μm; trace spacing = 15 μm; trace thickness = 4 μm; and impedance = 50.78 Ω, which is acceptable. 

### 5.2. Test Vehicle Verified by ANSYS

**Figure 9:**
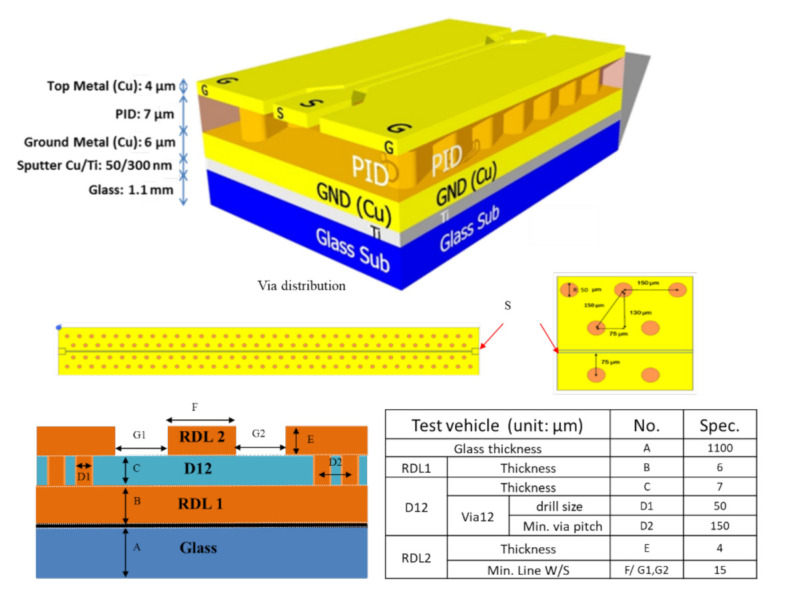
Detailed test vehicle designed by Polar.

Guided by the result of Polar, a detailed CPWG design is shown in Figure 9. It can be seen that: (a) glass thickness = 1.1 mm; (b) ground metal = 6 μm; (c) the PID = 7 μm; (d) the via size = 50 μm and minimum via pitch = 150 μm; (e) the top metal = 4 μm; (f) metal line width = 15 μm and line spacing = 15 μm; and (g) there are two different kinds of pad size: 50 and 80 μm. In this study, the specifications are: impedance = 50 ± 2.5 Ω; insertion loss (S_21_) > −3 dB; and return loss (S_11_) < −10 dB.

**Figure 10:**
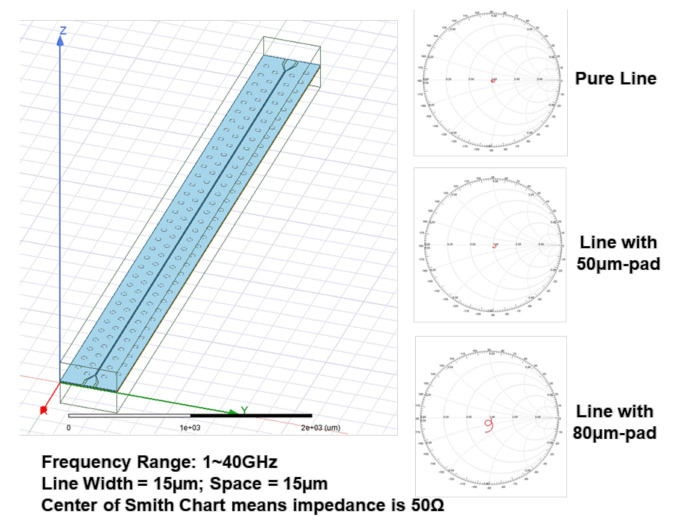
ANSYS HFSS model and Smith charts.

**Figure 11:**
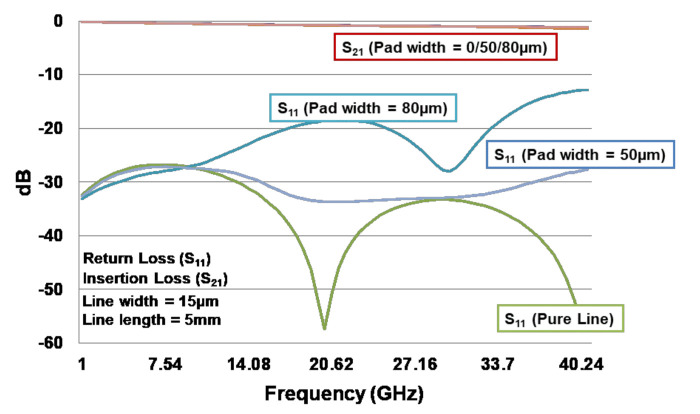
Return loss (S_11_) and insertion loss (S_21_) of test vehicle with Dk and Df from vendor 1 (ANSYS).

The model for ANSYS’ HFSS is shown in Figure 10, where the results (Smith charts) are also shown. It can be seen that, for the frequencies under consideration (1 to 40 GHz) and for the case of pure line, the impedance is 50 Ω, which confirms the design by Polar. The effect of the pad sizes (50 μm and 80 μm) for the transmission line measurement is to increase the impedance, as shown in Figure 11.

Figure 11 shows the return loss (S_11_) and insertion loss (S_21_) of the test vehicle with Dk and Df from vendor 1 and with different pad size for measurement purposes. It can be seen that the insertion loss is almost the same for the pad size = 0, 50, and 80 µm, and the values are larger than −3 dB, which is acceptable. On the other hand, the return loss is dependent on the pad size. In general, the smaller the pad size, the smaller the dBs of the return loss. Nevertheless, all their values are less than −10 dB, which meets the specification. Thus, this design will be fabricated for the cross-section analysis, TDR, and VNA measurements.

## 6. Test Vehicles Fabrication

**Figure 12:**
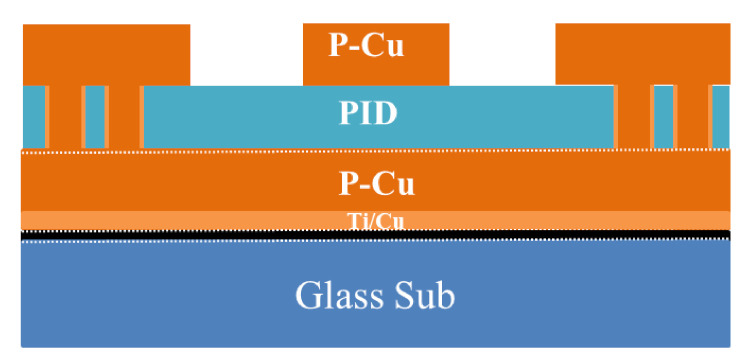
The schematic of test vehicle.

**Figure 13:**
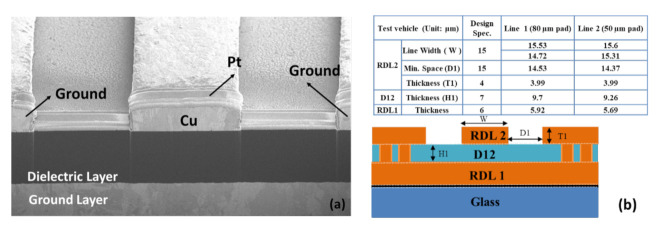
(**a**) The SEM image of test vehicle for TDR measurement and (**b**) detailed dimensions of actual test vehicle.

Figure 12 shows the schematic of the test vehicle. The key process steps are: after cleaning, first slit coat a released film on a glass carrier (515 mm × 510 mm × 1.1 mm) then PVD (physical vapor deposition) Ti/Cu (50/300 nm). It is followed by photoresist and laser direct imaging (LDI) and development then EDC (electrochemical deposition) Cu, photoresist striping, and Ti/Cu etching to form the Cu ground plane or RDL1. In order to spin coat the PID (photoimageable dielectric), the carrier is laser drilled (cut) into nine subpanels (150 mm × 150 mm). It is followed by laser drilling on the PID, sputtering Ti/Cu, spinning photoresist, LDI and development, EDC Cu, photoresist striping, and seed layer etching to form the Cu line or RDL2 (Figure 13).

**Figure 14:**
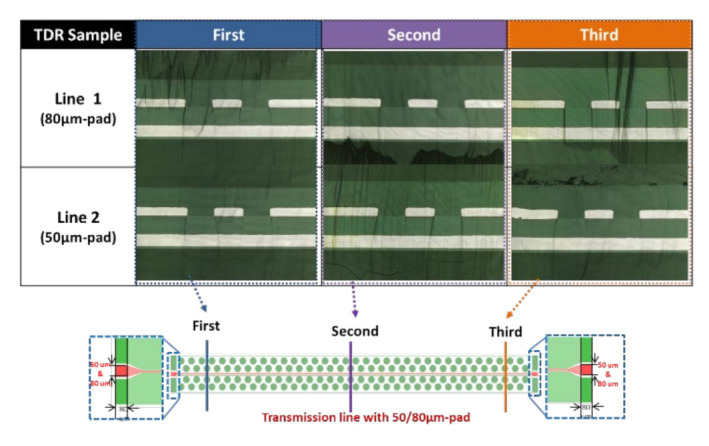
Cross-section images.

Figure 13a shows the scanning electric microscope (SEM) image of the test vehicle, and Figure 13b shows the detailed dimensions of the actual test vehicle. Figure 14 shows the cross sections. The average width, space, and thickness of the trace for both pads (50 and 80 µm) are, respectively, ~15 µm, ~15 µm, and ~4 µm. The thickness of the ground layer is about 6 µm. These values are close to the design specification. However, the thickness of the dielectric layer is 9.2 µm (50 µm pad width) and 9.7 µm (80 µm pad width). These values are > 30% higher than the specification. In addition, the Pt layer is only the pre-sputter protection layer, which reduces the charge effect during SEM observation.

## 7. TDR Measurement and Results

**Figure 15:**
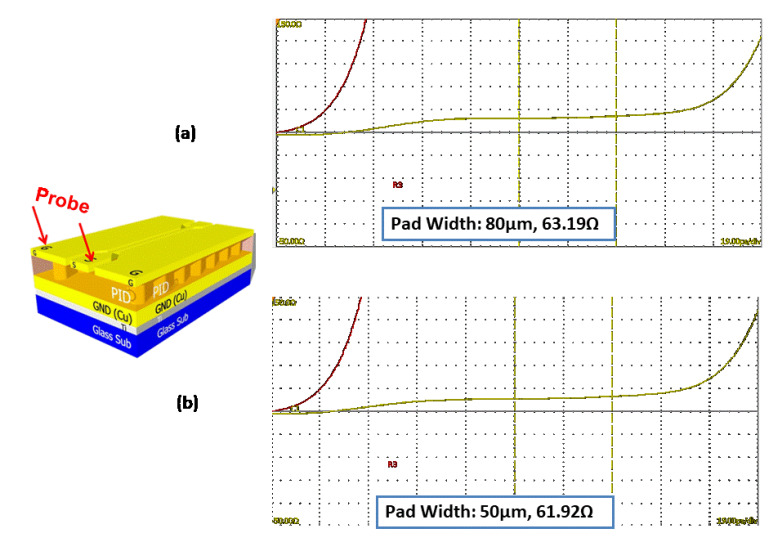
TDR measurement results. (**a**) For 80 μm pad width. (**b**) For 50 μm pad width.

The TDR measurement of the test structure is performed by the Oscilloscope TD8000/DSA8300 at 20 GHz. The test temperature is at room temperature (23 ± 2) °C. The line width is 15 µm, and the line length is 10 mm. The test pad sizes are 50 and 80 µm, respectively. The measurement results are shown in Figure 15. It can be seen that the impedance for pad width equal to 50 µm is 61.92 Ω, while for 80 µm, it is 63.19 Ω. These values are higher than that (~50 Ω) predicted by the Polar/ANSYS. This is due to the difference in the dielectric thickness between the design/analysis and the real structure. This also could be due to the variation of material properties (in design and simulation).

## 8. Effective Dielectric Constant (Ɛ_eff_)

**Figure 16:**
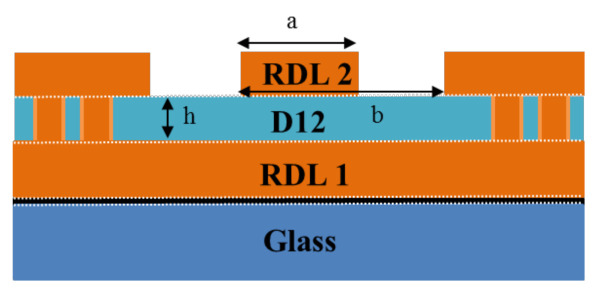
Definition of algebra in the impedance equation.

**Figure 17:**
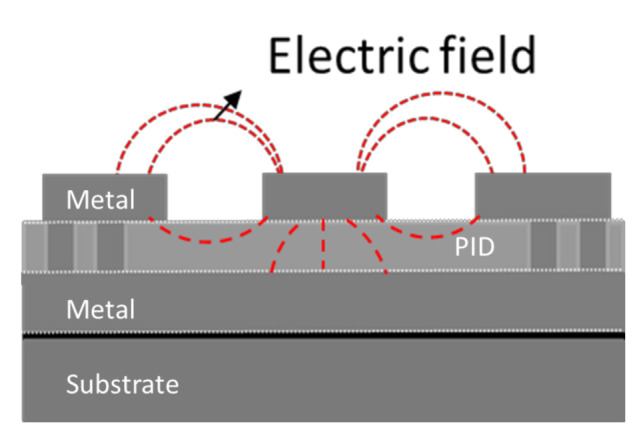
The schematic of GCPW with electric field.

The effective dielectric constant (Ɛ_eff_) provides a reference dielectric constant for design and simulation of complex material and/or stack structure. In this study, the effective Dk (Ɛ_eff_) of the test vehicle is calculated by a closed-form equation [19]. Figure 16 and Figure 17 show the definition of algebra in the impedance equation. It can be seen that Ɛ_eff_ = 2.19 for pad width = 80 μm and Ɛ_eff_ = 2.116 for pad width = 50 μm. These values are smaller than the measured value (~2.5) but are reasonably close.

Impedance Equation:

(1) $$Z_{0}=\frac{60.0\pi }{\sqrt{\varepsilon_{\mathrm{eff}}}}\frac{1.0}{\frac{K\left(k\right)}{K\left(k^{\prime }\right)}+\frac{K\left(k1\right)}{K\left(k1^{\prime }\right)}}$$

Re-write the equation:

(2) $$\varepsilon_{\mathrm{eff}}={\left(\frac{60\pi }{Z_{0}\times \left[\frac{K\left(k\right)}{K\left(k^{\prime }\right)}+\frac{K\left(k1\right)}{K\left(k1^{\prime }\right)}\right]}\right)}^{2}$$

where $k=\frac{a}{b},\;k^{\prime }=\sqrt{1.0-k^{2}},\;k1^{\prime }=\sqrt{1.0-k1^{2}}$, and $k=\frac{\tanh\left(\frac{\pi a}{4h}\right)}{\tanh\left(\frac{\pi b}{4h}\right)}$; “*a*” is the trace width, “*b*” is the sum of the track width plus the gaps either side, “*h*” is the height of the dielectric layer, shown in Figure 16.

Elliptical equation:

(3) $$K\left(k\right)=\frac{\pi }{2a_{n}}$$

where $a_{n}=\frac{a_{n-1}+b_{n-1}}{b},\;b_{n}=\sqrt{a_{n-1}-k^{2}},\;k1^{\prime }=\sqrt{1.0-k1^{2}}$, $k=\frac{\tanh\left(\frac{\pi a}{4h}\right)}{\tanh\left(\frac{\pi b}{4h}\right)}$ and *n* is iteration.

As shown in Figure 17, the electric field (red dashed line) travels across dielectric and air. In other words, the effective dielectric constant consists of the effects from fabrication and air in the study. Otherwise, the value of the dielectric constant may also be affected by the method of pre-treatment of the sample, measurement instrument, and measurement environment.

## 9. VNA Measurement and Correlation with Simulation Results

### 9.1. VNA Measurements

**Figure 18:**
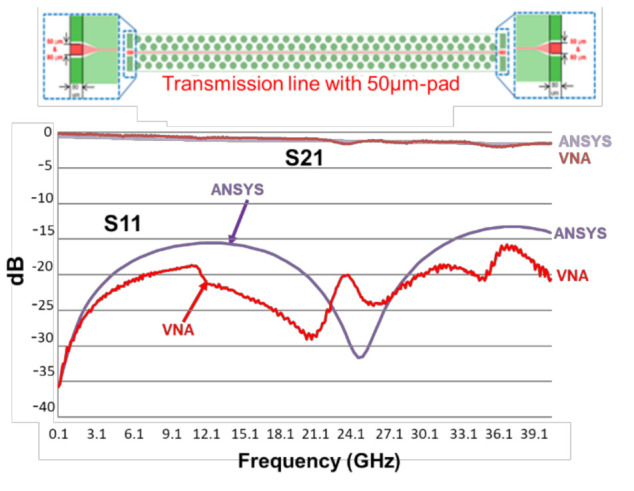
The VNA measurement results of test vehicle (50 μm pad) and correlation with ANSYS results.

**Figure 19:**
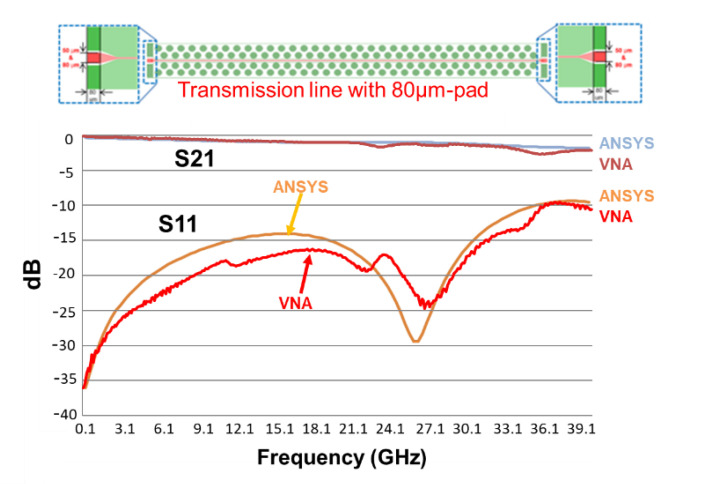
The VNA measurement results of test vehicle (80 μm pad) and correlation with ANSYS results.

**Figure 20:**
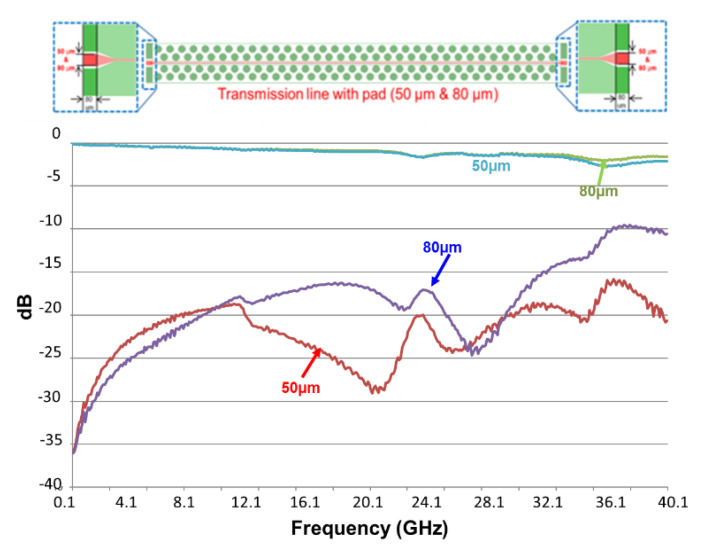
Comparison of VNA measurement results between the test vehicle with 50 μm pad and 80 μm pad.

The VNA (vector network analyzer) of the test vehicle is by Anritsu. The chuck size is 10 × 10 cm^2^, and it is measured at room temperature, 23 ± 2 °C. The designed line length and width are, respectively, 5 mm and 15 µm. The pad widths are 50 and 80 µm. The frequencies are from 1 to 67 GHz. Figure 18 and Figure 19 show the measurement results (up to 40 GHz). First of all, it can be seen that for both cases, S_21_ is greater than −3 dB, and S_11_ is less than −10 dB. For S_21_, the responses are not dependent on the pad width, except (with slight difference) at very high frequencies. On the other hand, for S_11_, the responses are dependent on the pad width; even the trends are basically the same. The one with 50 µm pad width performs better than the one with 80 µm, as shown in Figure 20.

### 9.2. Correlation of VNA Measurements with ANSYS Simulations

First of all, the simulation results (with PID = 7 μm) shown in Figure 11 cannot be used to compare with the VNA measurement results of the real structure (with PID > 9 μm). Further AYSYS/HFSS simulations with the real PID thicknesses—9.2 μm for 50 μm pad and 9.7 μm for 80 μm pad—are performed, and the results are shown in Figure 18 and Figure 19. It can be seen that the simulation results and the measurement results, in both the trend and magnitude, correlated very well.

## 10. Summary

➢ A systematic approach and complete flow to characterize the electrical performance (from 1 GHz to 40 GHz) of low-loss insulation materials by utilizing the FPOR, simulation, and S-parameter measurement have been provided. The Dk and Df of low-loss dielectric materials have been measured by the FPOR technique and, based on the IEC 61189:2015, a sample preparation produced. These values compared very well with those from the data sheets of the raw materials. It has been found that: (a) the BCB and PPE samples have a better performance in terms of the electrical material properties (Dk and Df) and repeatability; (b) the PI sample has the worst repeatability and electrical material properties; and (c) the Dk and Df measurement results are affected by environment, measuring instrument, and sample fabrication flow.
➢ Based on Polar and ANSYS’ HFSS, a CPWG test vehicle was designed and fabricated. The impedance of the test vehicle was measured by TDR, and the effective Dk of the test vehicle was calculated through a closed-form equation and the real dimensions of the metal line width, spacing, and thickness of the fabricated test vehicle. The insertion loss and return loss of the test vehicle were measured by VNA, and their trends and values correlated very well with the ANSYS’ HFSS simulation results based on the real dimension of the fabricated test vehicle. It was found that the thickness of the dielectric material (PID) plays a very important role in the electrical performance, such as the insertion loss and return loss. Thus, controlling the thickness of the PID is a very critical process step during manufacturing.
➢ The systematic approach to design, measurement, and simulation presented herein could be useful in design and/or manufacturing for high-speed and high-frequency applications.

## Figures and Tables

**Table 1:** Raw Materials Vendors and Their Data Sheets.

Items			Company		
			Vendor 1	Vendor 2	Vendor 3
Type		-	BCB	PPE	PI
Tone		-	Negative		
Curing	Temperature	°C	170/200	200	230
	Time	min	60/60	-	-
Developer			PGMEA	PGMEA	-
Electrical Properties	Dielectric constant (Dk)		2.66, 2.64	2.48, 2.57	3.07, 3.11, 2.9
	Dissipation factor (Df)		0.0031, 0.0032	0.003, 0.003	0.01, 0.01, 0.01
	Frequency (GHz)		28.3, 39.6	28, 40	19.36, 29.1, 38.9
Physical Properties	CTE α1 (<Tg)	ppm/K	31	60	-
	Tg	°C	170	215	-
	Moisture absorption	%	0.17 (23 °C/45%RH)	0.03 (23 °C/85%RH)	2.23 (23 °C/80%RH)
	Residual stress	MPa	20 @ 23 °C	14	-
	5% weight loss temp.	°C	340	413 @ N_2_	340
Mechanical Properties	Young’s modulus	GPa	2.4	1.6	3.9
	Elongation (RT)	%	13	35	62
	Tensile strength	Mpa	84	60	197
	Poisson’s ratio	-	0.36	-	-

**Table 2:** Sample Dimensions.

	Form	Vendor 1	Vendor 2	Vendor 3
Type	-	BCB	PPE	PI
Raw Material	Liquid			
Sample (Film)	Prapared by Unimicron	10 cm × 10 cm × 28 µm (UMTC 1)	10 cm × 10 cm × 57 µm (UMTC 2)	10 cm × 10 cm × 17 µm (UMTC 3)
	Prepared by Vendor	10 cm × 10 cm × 18 µm (Vendor 1)	10 cm × 10 cm × 30 µm (Vendor 2)	NA

**Table 3:** Dk and Df of Vendor 1.

	Samples/ Data Sheet	Frequency						
		21.3	25.5	28.2	32.4	35.2	38	40.7
Dk	UMTC 1 (Sample)	2.56	2.53	2.51	2.49	2.48	2.46	2.44
	Vendor 1 (Sample)	2.67	2.66	2.65	2.65	2.64	2.62	2.62
	Vendor 1 (Data sheet)	NA	NA	2.66 (28.3 GHz)	NA	NA	2.64 (39.6 GHz)	NA
Df	UMTC 1 (Sample)	0.0025	0.0033	0.0030	0.0029	0.0029	0.0034	0.0043
	Vendor 1 (Sample)	0.0016	0.0032	0.0033	0.0026	0.0041	0.0030	0.0035
	Vendor 1 (Data sheet)	NA	NA	0.0031 (28.3 GHz)	NA	NA	0.0032 (39.6 GHz)	NA

**Table 4:** Dk and Df of Vendor 2.

	Samples/ Data Sheet	Frequency (GHz)						
		21.3	25.5	28.2	32.4	35.2	38	40.7
Dk	UMTC 2 (Sample)	2.47	2.47	2.47	2.45	2.53	2.47	2.47
	Vendor 2 (Sample)	2.58	2.58	2.59	2.6	2.61	2.62	2.615
	Vendor 2 (Data sheet)	NA	NA	2.48 (28 GHz)	NA	NA	NA	2.57 (40 GHz)
Df	UMTC 2 (Sample)	0.0025	0.0026	0.0025	0.0025	0.0025	0.0026	0.0030
	Vendor 2 (Sample)	0.0016	0.0025	0.0028	0.0025	0.0034	0.0028	0.0032
	Vendor 2 (Data sheet)	NA	NA	0.003 (28.3 GHz)	NA	NA	NA	0.003 (40 GHz)

**Table 5:** Dk and Df of Vendor 3.

	Samples/ Data Sheet	Frequency (GHz)						
		21.3	25.5	28.2	32.4	35.2	38	40.7
Dk	UMTC 3 (Sample)	3.26	3.25	3.24	3.23	3.20	3.23	3.23
	Vendor 3 (Data sheet)	3.07 (19.36 GHz)	NA	3.11 (29.1 GHz)	NA	NA	NA	2.9 (38.9 GHz)
Df	UMTC 3 (Sample)	0.0119	0.0121	0.0127	0.0125	0.0122	0.0129	0.0136
	Vendor 3 (Data sheet)	0.01 (19.36 GHz)	NA	0.01 (29.1 GHz)	NA	NA	NA	0.01 (38.9 GHz)

## Data Availability

Not applicable.

## References

[1] Tasaki T. (2018). Low Transmission Loss Flexible Substrates using Low Dk/Df Polyimide Adhesives. TechConnect Briefs.

[2] Nishimura I., Fujitomi S., Yamashita Y., Kawashima N., Miyaki N. Development of new dielectric material to reduce transmission loss. Proceedings of the 2020 IEEE 70th Electronic Components and Technology Conference—ECTC.

[3] Yamamoto K., Koga S., Seino S., Higashita K., Hasebe K., Shiga E., Kida T., Yoshida S. Low Loss BT resin for substrates in 5G communication module. Proceedings of the 2020 IEEE 70th Electronic Components and Technology Conference—ECTC.

[4] Kakutani T., Okamoto D., Guan Z., Suzuki Y., Ali M., Watanabe A., Kathaperumal M., Swaminathan M. Advanced Low Loss Dielectric Material Reliability and Filter Characteristics at High Frequency for mmWave Applications. Proceedings of the 2020 IEEE 70th Electronic Components and Technology Conference—ECTC.

[5] Hayes C., Wang K., Bell R., Calabrese C., Kong J., Paik J., Wei L., Thompson K., Gallagher M., Barr R. Low Loss Photodielectric Materials for 5G HS/HF Applications. Proceedings of the International Symposium on Microelectronics.

[6] Hayes C., Wang K., Bell R., Calabrese C., Gallagher M., Thompson K., Barr R. High Aspect Ratio, High Resolution, and Broad Process Window Description of a Low Loss Photodielectric for 5G HS/HF Applications Using High and Low Numerical Aperture Photolithography Tools. Proceedings of the 2020 IEEE 70th Electronic Components and Technology Conference—ECTC.

[7] Han K., Akatsuka Y., Cordero J., Inagaki S., Nawrocki D. Novel Low Temperature Curable Photo-Patternable Low Dk/Df for Wafer Level Packaging (WLP). Proceedings of the 2020 IEEE 70th Electronic Components and Technology Conference—ECTC.

[8] Sato J., Teraki S., Yoshida M., Kondo H. High Performance Insulating Adhesive Film for High-Frequency Applications. Proceedings of the 2017 IEEE 67th Electronic Components and Technology Conference—ECTC.

[9] Ito H., Kanno K., Watanabe A., Tsuyuki R., Tatara R., Raj M., Tummala R. Advanced Low-Loss and High-Density Photosensitive Dielectric Material for RF/Millimeter-Wave Applications. Proceedings of the International Wafer Level Packaging Conference.

[10] Lau J.H. (2021). Semiconductor Advanced Packaging.

[11] Guo J., Wang H., Zhang C., Zhang Q., Yang H. (2020). MPPE/SEBS Composites with Low Dielectric Loss for High-Frequency Copper Clad Laminates Applications. Polymers.

[12] Vinnik D.A., Zhivulin V.E., Sherstyuk D.P., Starikov A.Y., Zuzina P.A., Gudkova S.A., Zherebtsov D.A., Rozanov K.N., Trukhanov S.V., Astapovich K.A. (2021). Electromagnetic properties of zinc-nickel ferrites in frequency range of 0.05–10 GHz. Mater. Today Chem..

[13] Zdorovets M.V., Kozlovskiy A.L., Shlimas D.I., Borgekov D.B. (2021). Phase transformations in FeCo-Fe_2_CoO_4_/Co_3_O_4_-spinel nanostructures as a result of thermal annealing and their practical application. J. Mater. Sci. Mater. Electron..

[14] Almessiere M.A., Güner S., Slimani Y., Hassan M., Baykal A., Gondal M.A., Baig U., Trukhanov S.V., Trukhanov A.V. (2021). Structural and magnetic properties of Co_0.5_Ni_0.5_Ga_0.01_Gd_0.01_Fe_1.98_O_4_/ZnFe_2_O_4_ spinel ferrite nanocomposites: Comparative study between sol-gel and pulsed laser ablation in liquid approaches. Nanomaterials.

[15] Kozlovskiy A.L., Shlimas D.I., Zdorovets M.V. (2021). Synthesis, structural properties and shielding efficiency of glasses based on TeO_2_-(1-x)ZnO-xSm_2_O_3_. J. Mater. Sci. Mater. Electron..

[16] Araki H., Kiuchi Y., Shimada A., Ogasawara H., Jukei M., Tomikawa M. (2020). Low Df Polyimide with Photosenditivity for High Frequency Applications. J. Photopolym. Sci. Technol..

[17] Araki H., Kiuchi Y., Shimada A., Ogasawara H., Jukei M., Tomikawa M. Low Permittivity and Dielectric Loss Polyimide with Patternability for High Frequency Applications. Proceedings of the 2020 IEEE 70th Electronic Components and Technology Conference—ECTC.

[18] Karpisz T., Salski B., Kopyt P., Krupka J. (2019). Measurement of Dielectrics From 20 to 50 GHz With a Fabry–Pérot Open Resonator. IEEE Trans. Microw. Theory Tech..

[19] Wadell B. (1991). Transmission Line Design Handbook.

